# Supplementary material for: Comparative multiomics analysis of cell physiological state after culture in a basket bioreactor
Source: Sci Rep. 2022 Nov 23;12:20161. doi: 10.1038/s41598-022-24687-4 (PMC9686226; doi:10.1038/s41598-022-24687-4)

4039:5994209:-@NW_023666042.1:5993360:5993545:-@NW_023666042.1:5991254:5991448:-@NW_023666042.

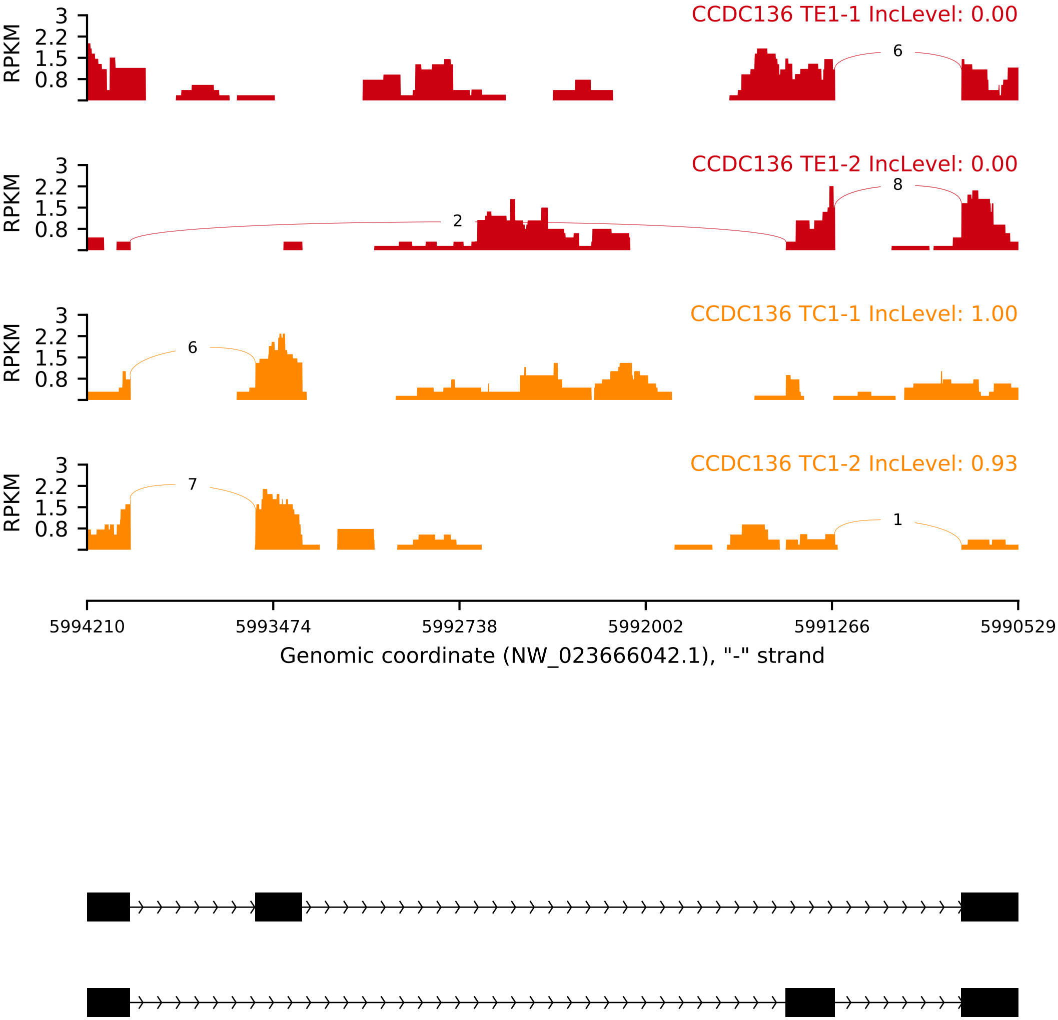

Supplement: Supplementary file 1 — Supplementary Information 1. [file 41598_2022_24687_MOESM1_ESM.zip › raw data/Transcriptomic raw data/7.AS/2.dasplot/TE1vsTC1/MXE/1_CCDC136_NW_023666042.1_5994039_5994209_-@NW_023666042.1_5993360_5993545_-@NW_023666042.1_5991254_5991448_-@NW_023666042.1_5990527_5990754_-.pdf]
